# Depression and anxiety in early adulthood following adolescent onset disordered eating: a longitudinal study

**DOI:** 10.1186/2050-2974-2-S1-O57

**Published:** 2014-11-24

**Authors:** Linda Varcoe, Ross King, Craig Olsen

**Affiliations:** 1 School of Psychology, Deakin University, Melbourne, Australia; 2 Murdoch Children's Research Institute, Melbourne, Australia

## 

Disordered eating is common among adolescent females. Clinical eating disorders are associated with high rates of depression and anxiety in adulthood. However, the extent to which this is the case for subthreshold disordered eating remains unclear, and raises issues for early detection and intervention, prognosis and treatment planning. The current study aimed to enhance knowledge on the longitudinal course of adolescent onset disordered eating and young adult psychopathology (specifically depression and anxiety); and to establish whether the outcomes differed among males and females. Longitudinal data from a large community sample, the Australian Temperament Project (ATP) was utilized, with respect to disordered eating staus during adolescence and mood and anxiety symptoms in early adulthood (N = 686). Overall, adolescents meeting the criteria for subthreshold disordered eating have higher levels of anxiety in adulthood compared to the non-disordered eating group. Furthermore, males with disordered eating experienced higher levels of depression at eight-year follow up. Given the level of subsequent psychopathology associated with adolescent onset disordered eating, targeting preventative interventions at adolescents with subthreshold disordered eating may reduce the risk of developing a clinical eating disorder as well as reducing the risk of developing depression and anxiety in early adulthood.

This abstract was presented in the **Disordered Eating and Body Image** stream of the 2014 ANZAED Conference.

